# Micropropagation of *Andrographis producta* through axillary and adventitious shoot regeneration

**DOI:** 10.1186/s43141-022-00438-w

**Published:** 2022-11-01

**Authors:** Sathish Shekhappa Kadapatti, Hosakatte Niranjana Murthy

**Affiliations:** Department of Botany, Karnatak University, Dharwad, 580003 India

**Keywords:** Adventitious shoots, *Andrographis producta*, Axillary shoots, *In vitro* regeneration

## Abstract

**Background:**

*Andrographis producta* (C. B. Clarke) Gamble is a valuable medicinal plant that yields several therapeutic compounds. In addition, this species is endemic to the Western Ghats regions of South India. Natural populations of *Andrographis producta* have dwindled due to the overexploitation of this species. The objective of the present study was to develop a reliable *In vitro* propagation protocol for this plant species.

**Results:**

*In vitro* plant regeneration protocol has been developed in *Andrographis producta* using nodal and shoot tip explants. The highest axillary shoots (14.60) were regenerated from nodal explants on MS medium amended with 10 μM 2-iP. Similarly, on MS media amended with 5 μM BAP, 17.50 shoots were regenerated from shoot tip explants. Optimal of 27.66 adventitious shoots were regenerated from the cut end of shoot tip explants on MS medium amended with 10 μM 2-iP. Medium amended with 10 μM 2-iP was optimum for regeneration of multiple axillary shoots from nodal explants and for the adventitious shoots regeneration from shoot tip explants. Shoot tips were ideal explants for the micropropagation of *A*. *producta*. Qarter sthrength MS media supplemented with 10 μM IBA has resulted in maximum rooting of the shoots.

**Conclusions:**

A reliable *In vitro* micropropagation method was developed in *Andrographis producta* through direct organogenesis, and this method is helpful for the multiplication, conservation, and utilization of this plant.

## Background

*Andrographis* (Acanthaceae) comprises threatened and endemic medicinal plants such as *A. producta*, *A. alata*, *A. lineata*, and *A. echioides* distributed in the South Indian region [1–4]. In contrast, the *A. paniculata* is a regularly grown plant in India. It has been produced in Southeast Asian countries like China, India, and Malaysia to meet the demand for both traditional and modern medicinal systems [5]. *Andrographis* species contain diterpenoids, flavonoids, and xanthones which are reported to possess a wide range of biological properties such as anticancer, antidiabetic, anti-inflammatory, antimicrobial, antioxidant, immunostimulant, and hepatoprotective activities [6].

*Andrographis producta* (C. B. Clarke) Gamble is an erect herb that is endemic to the Western Ghats in India [1]. *Andrographis producta* is reported to possess phytochemicals, including phenolics, flavonoids, and organic acids, which have shown potent antioxidant activities [7]. Gas chromatography and mass-spectroscopic analysis have demonstrated the presence of various bioactive compounds such as 2-methoxy-4-vinylphenol, 2,4-ditert-butyl-phenol, phytol, 5-hydroxy-7,8-dimethoxyflavone, gammasitosterol, salvigenin, solanesol, and alpha-terpinene in this plant [7]. *Andrographis producta* is traditionally used to treat various health ailments, including intestinal worms, relieve constipation, eliminate phlegm in womens during postpartum by native people [7], and treat skin diseases by the local tribes of Nilgiris Biosphere Reserve [8]. Endemism, habitat loss, forest fires, and overexploitation are significant threats to the survival of *Andrographis* species [9]. Therefore, plant regeneration protocols have been developed for *A*. *paniculata* [10], *A*. *alata* [11], *A*. *macrobotrys* [12], *A*. *echioides* [13], and *A*. *lineata* [14]. In addition to the facts mentioned above, conventional propagation of *Andrographis producta* through seeds is hampered by poor seed germination and short seed viability [15, 16], and there are no regeneration protocols for the micropropagation of *Andrographis producta*. Given the above, the *In vitro* propagation method was adopted for the large-scale production of *Andrographis producta* plants. Here, we report successful methods for large-scale propagation using nodal and shoot tip explants.

## Methods

### Plant material

Plants of *Andrographis producta* (C.B. Clarke) Gamble were collected from Bababudan Hill ranges, Chikamagalur district, Karnataka, India (lat: 13° 25′ 10.2108″; long: 75° 44′ 37.0026″; *MSL* 1467.30 m) and were maintained in a botanical garden*.* Identification of plant species was confirmed by Prof. S. R. Yadav, Shivaji University, Kolhapur, India, and voucher specimens were maintained at herbarium, Shivaji University, Kolhapur, India.

### *In vitro* seed germination

Seeds were sterilized with 5% (v/v) sodium hypochlorite solution for 15 min, cultured on 1/10th-strength Murashige and Skoog [17] (MS) medium supplemented with 3% sucrose, and solidified with 0.8% agar and incubated in culture room at 25 ± 2 °C with a photoperiod of 16/8 h (light and dark). All processes were carried out under sterile conditions using a laminar air-flow hood. In addition, the pH of the medium was set at 5.8 and sterilized by autoclaving at 121 °C for 15 min.

### Shoot tip and nodal cultures

Shoot tips (1–3 mm) and nodal explants (5 mm in length) were obtained from 6-week-old seedlings. They were cultured on MS nutrient medium supplemented with 3% (w/v) sucrose and 2.5, 5.0, 7.5, and 10.0 µM BAP, KN, 2-iP, and TDZ (HiMedia, India) individually. The cultures were maintained in culture room wherein temperature, light, and relative humidity were set at 25 ± 2 °C, 16 h light (50 µmol m^−2^ s^−1^)/8 h dark, and 60% relative humidity, respectively.

### *In vitro* root formation

Regenerated shoots were individually cultured onto ¼ strength MS nutrient medium containing 3% (w/v) sugar, supplemented with 1.0, 2.0, 5.0, and 10 µM IAA, IBA, and NAA (HiMedia, India) for induction of roots.

### Acclimatization of plants

Micropropagated plants (5 cm in height) were transplanted to pots containing equal volumes of cocopeat and vermiculite and plants were reared in growth chambers wherein temperature, light, and relative humidity were set at 25 ± 2 °C, 16 h light (50 µmol m^−2^ s^−1^)/8 h dark, and 60%, respectively. After 2 weeks, plants were transferred to potting mix containing soil and cocopeat and maintained in the greenhouse.

### Histological analysis

For histological studies, cultured nodal and shoot tip explants were fixed in FAA (10 ml of formalin, 85 ml of 70% ethyl alcohol, and 5 ml of glacial acetic acid) for 12 h at room temperature and dehydrated by ethanol-butyl alcohol series and embedded in parafilm as recommended by Johansen [18]. The material was sectioned (thickness of 6 µm) and stained with 0.05% toluidine blue (HiMedia, Mumbai, India) and examined under a compound microscope (Nikon, Tokyo, Japan).

### Data analysis

A randomized block method was followed for the establishment of experiments. Data were statistically analyzed with the help of one-way analysis of variance (ANOVA) followed by Duncan’s multiple range test was applied using SPSS statistical software (version 20).

## Results

### Axillary shoot regeneration from nodal explants

**Fig. 1:**
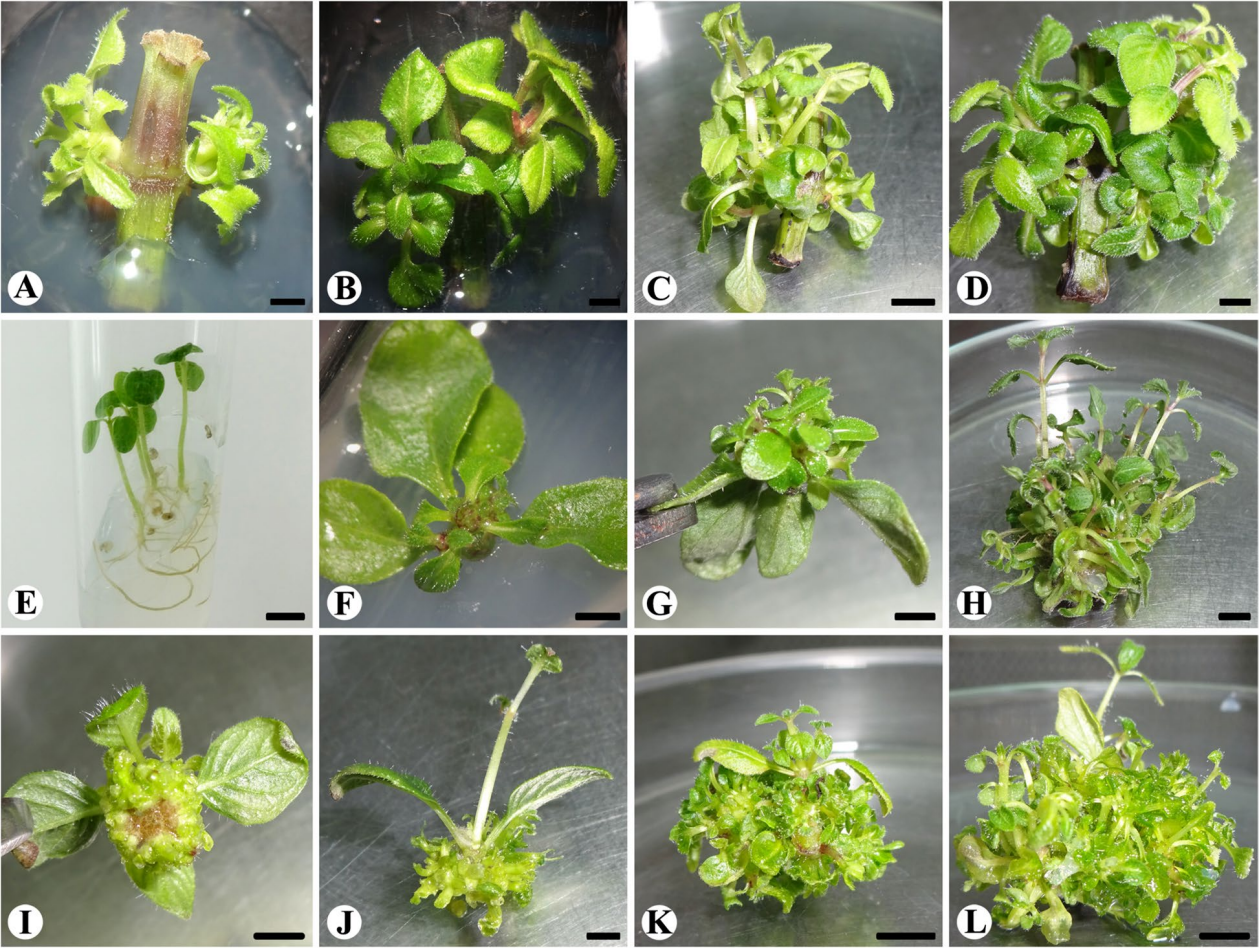
*In vitro* shoot regeneration from nodal and shoot tip explants of *Andrographis producta* on Murashige and Skoog medium supplemented with cytokinins. **A** Induction of axillary shoots from the nodal explant on MS medium supplemented with 10 μM 2-iP after 2 weeks of culture (bar-, 0.20 cm). **B** Shoot multiplication from nodal explant on MS medium supplemented with 10 μM 2-iP after 4 weeks (bar-, 0.23 cm). **C** Shoot proliferation from nodal explant on MS medium containing 10 μM 2-iP after 6 weeks (bar-, 0.35 cm). **D** Multiple shoots regeneration from nodal explant after 8 weeks of culture on medium supplemented with 10 μM 2-iP (bar-, 0.30 cm). **E** *In vitro* seed germination on 1/10th strength MS basal medium (bar-, 0.7 cm). **F** Initiation of multiple shoots from shoot tip explant on MS medium supplemented with 5 μM BAP after 2 weeks (bar-, 0.75 cm). **G** Proliferation of shoots from shoot tip explant on medium supplemented with 5 μM BAP after 4 weeks (bar-, 0.9 cm). **H** Multiple shoots regeneration from shoot tip explant after 8 weeks of culture on MS medium supplemented with 5 μM BAP (bar-, 0.98 cm). **I** Initiation of direct shoot buds from cut end region (basal region) of shoot tip explant after 2 weeks of culture on MS medium containing 10 μM 2-iP (bar-, 0.60 cm). **J** and **K** Proliferation of shoots from cut end region (basal region) on shoot tip explant on MS medium containing 10 μM 2-iP after 4 and 6 weeks, respectively (bar-, 0.55 cm and 1.15 cm for **J** and **K**, respectively). **L** Multiple shoots regenerated from a cut end region of shoot tip explant after 8 weeks of culture on MS medium supplemented with 10 μM 2-iP (bar, -1.03 cm)

**Fig. 2:**
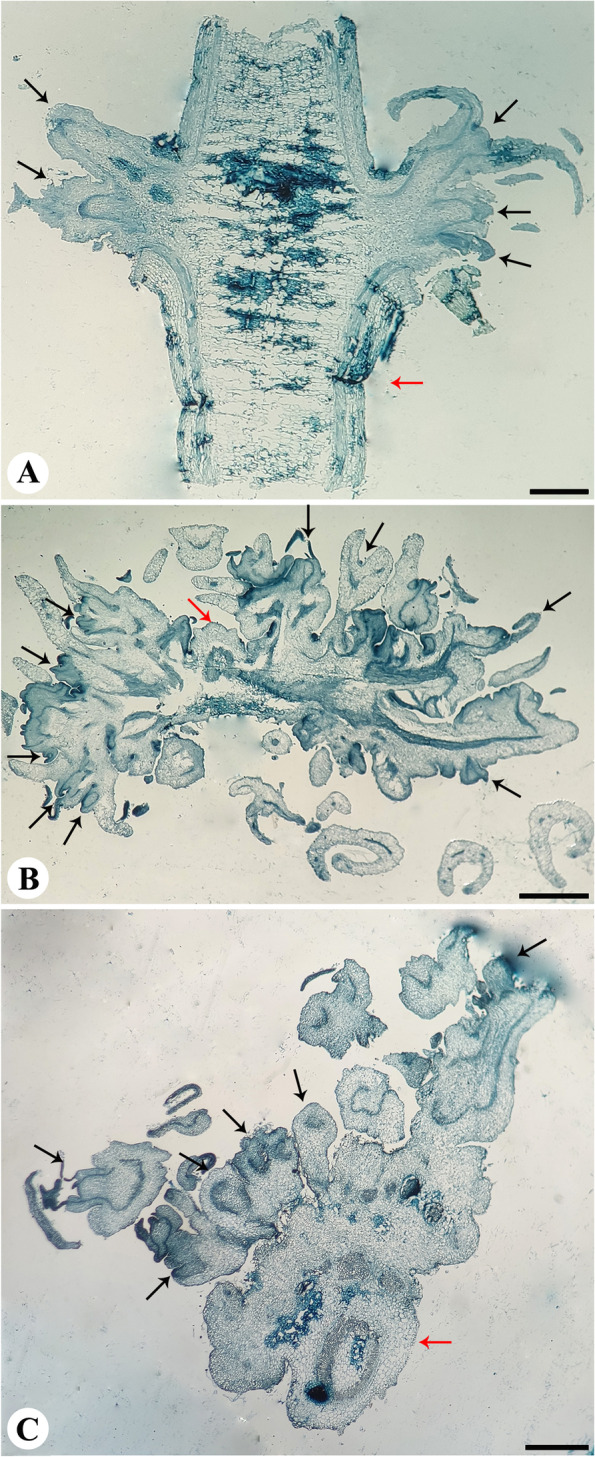
Histological studies on shoot regeneration from varied explants of *Andrographis producta*. **A** Longitudinal section of the nodal explant developed on MS medium with 10 μM 2-iP after 2 weeks of culture showing direct shoot regeneration (bar-, 0.075 cm). **B** Section through 4-week-old shoot tip cultured on MS medium containing 5 μM BAP showing multiple shoot regeneration (bar-, 0.112 cm). **C** Transverse section of 4-weeks-old shoot tip explant cultured on 10 μM 2-iP showing direct shoot induction (bar-, 0.075 cm) (Black- colored arrows indicate newly formed shoot buds and shoot, and red arrows indicate original explants)

The nodal explants cultured on MS medium containing cytokinin involved in axillary shoot induction within 2 weeks of culture (Table 1). Axillary shoots were regenerated on all cytokinin-supplemented media; however, optimum regeneration was observed on MS medium supplemented with 2-iP. The highest percentage of response, and a greater number of shoots, and mean shoot length were optimum with 2-iP containing medium (Table 1). On 2-iP containing media, initially, few shoots were emerged from nodal regions after 2 weeks in culture (Fig. 1A). Shoot proliferation was evident with the advancement of time (after 4 and 6 weeks) in culture (Table 1, Fig. 1 B–C); at the end of 8 weeks in culture, highest shoot proliferation was recorded (Fig. 1D). On MS media amended with 10 μM 2-iP, the highest number of 14.60 shoots were regenerated from nodal explants (Table 1). Nodal explants involved in direct shoot regeneration without callus phase or callus mediation were examined histologically (Fig. 2A).

**Table 1:** Influence of cytokinins supplemented to Murashige and Skoog medium on *In vitro* regeneration of shoots from *Andrographis producta* using nodal and shoot tip explants

Growth regulator (μM)	Percentage of response		Mean number of shoots per explant		Mean shoot length (cm)	
Control	Node 66.66	Shoot tip 83.33	Node 1.25 ± 0.25d	Shoot tip 1.00 ± 0.00f	Node 0.85 ± 0.13fgh	Shoot tip 1.32 ± 0.13e
KN						
2.5	83.33	33.33	1.60 ± 0.24d	1.00 ± 0.00f	1.79 ± 0.14a	1.50 ± 0.00de
5.0	66.66	50	1.25 ± 0.25d	1.00 ± 0.00f	0.70 ± 0.07gh	1.53 ± 0.14de
7.5	50	66.66	1.00 ± 0.00d	1.00 ± 0.00f	0.80 ± 0.05gh	1.30 ± 0.17e
10	50	50	1.00 ± 0.00d	1.00 ± 0.00f	0.60 ± 0.10 h	1.76 ± 0.03 cd
BAP						
2.5	66.66	83.33	2.25 ± 0.25d	10.40 ± 0.50e	0.71 ± 0.04gh	2.23 ± 0.12ab
5.0	66.66	100	5.75 ± 0.62c	17.50 ± 0.88c	0.90 ± 0.12efgh	2.45 ± 0.08a
7.5	83.33	83.33	7.00 ± 0.83c	14.00 ± 0.54d	1.03 ± 0.06cdefg	1.97 ± 0.08bc
10	83.33	83.33	9.60 ± 0.50b	10.00 ± 0.77e	1.35 ± 0.04bc	1.76 ± 0.05 cd
TDZ						
2.5	83.33	50	1.40 ± 0.24d	1.00 ± 0.00f	0.94 ± 0.08defgh	0.90 ± 0.05f
5	50	50	1.00 ± 0.00d	1.00 ± 0.00f	0.86 ± 0.06fgh	0.86 ± 0.06f
7.5	50	33.33	1.00 ± 0.00d	1.00 ± 0.00f	0.76 ± 0.08gh	1.20 ± 0.10ef
10	50	33.33	1.00 ± 0.00d	1.00 ± 0.00f	1.16 ± 0.08bcdef	1.30 ± 0.10e
2-iP						
2.5	66.66	100	2.00 ± 0.00d	16.16 ± 0.65 cd	1.22 ± 0.21bcde	2.11 ± 0.10abc
5.0	83.33	100	6.80 ± 0.80c	20.50 ± 1.02b	0.94 ± 0.06defgh	2.06 ± 0.12abc
7.5	83.33	100	9.20 ± 0.58b	21.50 ± 0.50b	1.24 ± 0.07bcd	2.03 ± 0.14bc
10	83.33	100	14.60 ± 0.74a	27.66 ± 1.05a	1.40 ± 0.08b	1.90 ± 0.07bcd

Mean data was recorded after 8 weeks of culture. Each value represents the mean ± standard error. Treatment means followed by different letters in their superscript are significantly different from each other (*p* = 0.05) according to Duncan’s multiple range test

### Multiple and adventitious shoot regeneration from shoot tip explants

Seeds were germinated on 1/10th strength MS basal medium (Fig. 1E), and shoot tips harvested from young seedlings were used for plant regeneration. Shoot tip explants cultured on MS medium amended with KN and TDZ (2.5, 5.0, 7.5, and 10 μM) developed a single shoot. However, the shoot tip explants cultured on BAP and 2-iP media showed a differential response. On media supplemented with BAP, shoot tip explants developed multiple shoots from shoot meristem (Table 1). Shoot tip explants initially produced a few shoots (Fig. 1F), and shoot proliferation was observed after 4 and 8 weeks in culture (Table 1; Fig. 1G–H). On MS media amended with 5 μM BAP, 100% shoot tips were responded and developed 17.50 shoots per explant (Table 1; Fig. 1H). Shoot tip explants which were cultured on 2-iP containing media showed adventitious shoot regeneration from the cut end of shoot tips. In contrast, a single shoot was regenerated from the shoot meristem on basal medium. Shoot buds sprouted from the cut end of the shoot tip on MS medium containing 10 μM 2-iP after 2 weeks in culture (Fig. 1I); such shoot buds were involved in proliferation in subsequent weeks (Fig. 1J–K). Optimal of 27.66 adventitious shoots were regenerated from the cut end of shoot tip explants on MS medium amended with 10 μM 2-iP (Table 1). Preexisting meristem of shoot tip explants cultured on 5 μM BAP divided and differentiated into multiple shoots, and shoot meristems directly generated multiple shoots without callus tissue’s mediation, according to histological preparations of shoot tip explants (Fig. 2B). Similar to this, histological studies revealed direct adventitious shoot regeneration (without callus intervention) from the cut end of shoot tip explants as a result of mitotic activity of epidermal cells in response to the media supplemented with 10 μM 2-iP (Fig. 2C).

### In vitro root formation

**Fig. 3:**
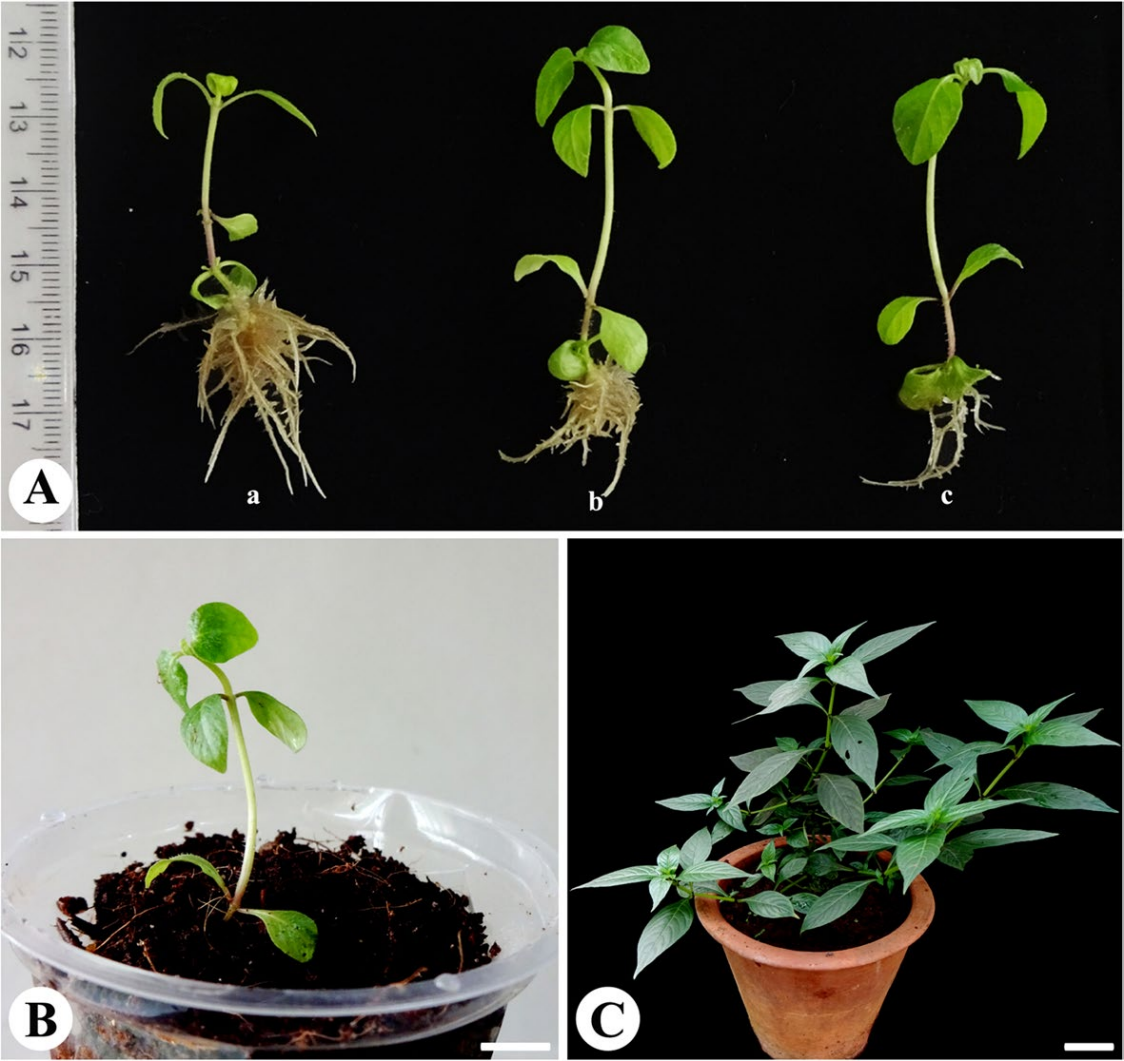
*In vitro* rooting of shoots and acclimatization of regenerated plants of *Andrographis producta*. **A** Induction of roots from shoots on quarter strength MS medium supplemented with 10 μM IBA (a), 5 μM IBA (b), and 1 μM IBA (c). **B** Acclimatization of regenerated plantlets in plastic cups containing soil and cocopeat (bar-, 0.85 cm). **C** Three- months -old greenhouse-grown plants in the pot containing soil, sand, and farmyard manure (bar-, 5.25 cm)

For root induction, *In vitro* raised shoots (2-5 cm in length) were cultured on quarter strength MS media amended with 1, 2, 5, and 10 μM IAA, NAA, or IBA, and the results are presented in Table 2. Roots were sprouted from the shoots on all the auxin-supplemented media (Table 2); however, optimal root induction was recorded on MS medium containing IBA. There was a linear increase in the number of roots with the increasing concentration of IBA in the medium (Table 2, Fig. 3A). The highest percent of root induction (100%) and optimum roots per shoot (18.66 per shoot) were observed on MS medium supplemented 10 μM IBA (Table 2, Fig. 3A).

**Table 2:** Influence of auxins supplemented to quarter strength of Murashige and Skoog medium on induction of roots from shoots of *Andrographis producta*

Growth regulator	Concentration of hormone (μM)	Percentage of response	Mean number of roots per shoot	Mean root length (cm)
Control	0	66.66	3.25 ± 0.47ij	2.47 ± 0.12c
IAA	1	50	2.33 ± 0.33j	1.96 ± 0.08d
	2	83.33	5.20 ± 0.37gh	3.70 ± 0.15a
	5	100	6.33 ± 0.42fgh	2.95 ± 0.08b
	10	100	8.50 ± 0.42de	2.60 ± 0.11c
NAA	1	100	13.00 ± 0.85b	2.01 ± 0.15d
	2	100	10.00 ± 0.51 cd	1.31 ± 0.12e
	5	83.33	10.40 ± 0.50c	1.24 ± 0.08e
	10	66.66	6.75 ± 0.62efg	1.87 ± 0.11d
IBA	1	83.33	4.80 ± 0.37hi	1.88 ± 0.11d
	2	100	7.50 ± 0.34ef	1.98 ± 0.10d
	5	100	9.83 ± 0.47 cd	1.30 ± 0.05e
	10	100	18.66 ± 1.02a	2.03 ± 0.09d

Mean data was recorded after 4 weeks of culture. Mean values followed by the same letter are not significantly different according to Duncan’s multiple range test (*p* = 0.05)

### Acclimatization

*In vitro* regenerated plantlets were removed from culture vessels and media adhering to the roots of the plantlets was carefully washed with distilled water. The plantlets were transplanted to poly-cups containing cocopeat and vermiculite (1:1 ratio) and reared in controlled conditions for 2 weeks (Fig. 3B). Later, they were transferred to bigger pots containing potting mix (Fig. 3C), and the survival percentage was 95%.

## Discussion

The present *In vitro* propagation studies reveal that multiple axillary shoots could be regenerated by using nodal explants of *A. producta* on MS medium supplemented with 2-iP. In contrast, media supplemented with KN, BAP, and TDZ were less efficient in multiple axillary shoot regeneration. On MS media amended with 10 μM 2-iP, the highest number of multiple shoots were regenerated from nodal explants. Additionally, histological examination showed that nodal explants directly produced shoots without the need for callus mediation (Fig. 2A), and the similar reports on use of histological evidences to trace the mode of regeneration from nodal explants have been reported in *Vitex trifolia* [23], and *Andrographis paniculata* [10], where meristematic cells at outer protoderm layer of axillary bud divided and differentiated into axillary shoots. Multiple axillary shoot regeneration from nodal explant on cytokinin supplemented medium was also reported in *Andrographis paniculata*, *Artemisia nilagirica* var. *nilagirica*, *Artemisia japonica*, *Feronia limonia*, *Nothapodytes nimmoniana*, *Spilanthes oleracea*, and *Vitex trifolia* [10, 19–24]. Among various cytokinins used in the present study, 2-iP supplemented medium was superior in axillary shoot induction. In contrast to the present results, BAP was reported to be potent cytokinin in axillary shoot induction in *Andrographis alata* and *A. macrobotrys* [11, 12].

Shoot tip explants of *Andrographis producta* developed multiple shoots from shoot meristem on MS media amended with 5 μM BAP. In contrast, the development of adventitious shoots was recorded from the cut end of the shoot tip explants (the basal portion of the shoot tips) on media amended with 2-iP. Optimal of 27.66 adventitious shoots were regenerated from the cut end of shoot tip explants on MS medium amended with 10 μM 2-iP. Histological preparations again showed direct regeneration of multiple shoots from the shoot meristem and basal portion of the shoot tips, and this is in consistent with the reports on *Clementis* cultivar where meristems of shoot tip are involved in division and differentiation to form direct shoots [25] and *Neolamarckia cadamba* [26], where epidermal and subepidermal cells regained mitotic activity and differentiated directly into new shoots. Among the four individual cytokinins tested, i.e., KN, BAP, TDZ, and 2-iP, the highest multiple shoot regeneration was achieved on 2-iP supplemented medium. Similar to the current results, an efficient *In vitro* plant regeneration was achieved from shoot tips explants of *Curculigo latifolia* [27] and *Enicostema axillare* [28] on TDZ and BAP supplemented medium, respectively. Adventitious shoot regeneration from shoot tip explants was described in *Vanda coerulea* by Jitsopakul et al. [29] which was the efficient mode of *In vitro* regeneration.

An essential stage for plantlet regeneration and adaptation is the rooting of an *In vitro* regenerated shoot. A single shoot of *Andrographis producta* cultivated on quarter strength MS media supplemented with 10 M IBA resulted in the optimum root induction. After 2 weeks of root initiation, there was a rapid root elongation. *Andrographis paniculata* [10], *Andrographis alata* [11], and *Andrographis macrobotrys* [12] all showed comparable results.

## Conclusion

The MS media with 10 μM 2-iP was proved as most effective for the adventitious shoot and axillary shoot induction from shoot tip and nodal explants, respectively. For rooting, qurater sthrength MS media amended with 10 μM IBA has resulted in maximum rooting. This study showed an efficient direct shoot regeneration of *Andrographis producta* using nodal and shoot tip explants. The *In vitro* regeneration protocol developed for *Andrographis producta* is useful for the multiplication and conservation of this plant.

## Data Availability

Not applicable.

## Abbreviations

BAP: 6-Benzylaminopurine
IAA: Indole-3-acetic acid
IBA: Indole-3-butyric acid
NAA: α-Naphthalene acetic acid
KN: Kinetin
2-IP: 2-Isopentenyl adenine
MS: Murashige and Skoog medium
TDZ: Thidiazuron
